# A Weighted Deep Representation Learning Model for Imbalanced Fault Diagnosis in Cyber-Physical Systems

**DOI:** 10.3390/s18041096

**Published:** 2018-04-05

**Authors:** Zhenyu Wu, Yang Guo, Wenfang Lin, Shuyang Yu, Yang Ji

**Affiliations:** 1 Engineering Research Center of Information Network, Ministry of Education, Beijing University of Posts and Telecommunications, Beijing 100876, China; ji.yang.0001@gmail.com; 2 Key Laboratory of Universal Wireless Communications, Ministry of Education, Beijing University of Posts and Telecommunications, Beijing 100876, China; guoyang.bupt@gmail.com (Y.G.); linwenfang@bupt.edu.cn (W.L.); yushuyanggyx@163.com (S.Y.)

**Keywords:** PHM, deep learning, feature extraction, time series, class imbalance, fault diagnosis, cyber-physical system

## Abstract

Predictive maintenance plays an important role in modern Cyber-Physical Systems (CPSs) and data-driven methods have been a worthwhile direction for Prognostics Health Management (PHM). However, two main challenges have significant influences on the traditional fault diagnostic models: one is that extracting hand-crafted features from multi-dimensional sensors with internal dependencies depends too much on expertise knowledge; the other is that imbalance pervasively exists among faulty and normal samples. As deep learning models have proved to be good methods for automatic feature extraction, the objective of this paper is to study an optimized deep learning model for imbalanced fault diagnosis for CPSs. Thus, this paper proposes a weighted Long Recurrent Convolutional LSTM model with sampling policy (wLRCL-D) to deal with these challenges. The model consists of 2-layer CNNs, 2-layer inner LSTMs and 2-Layer outer LSTMs, with under-sampling policy and weighted cost-sensitive loss function. Experiments are conducted on PHM 2015 challenge datasets, and the results show that wLRCL-D outperforms other baseline methods.

## 1. Introduction

Predictive maintenance plays a critical role in Cyber-Physical Systems (CPSs) since a large amount of sensor data are being collected for monitoring the operational behavior of machines. Consequently, there is increasing interest in exploiting data from sensors for fault diagnostics and prognostics. A robust and accurate failure prognostic system helps prevent fatal accidents, saves costs and increases manufacturing efficiency [1]. Industrial data have two main characteristics: one is multi-dimensionality in which several sensor channels are monitored as a representation of the working states; the other is class-imbalanced in which faulty data are significantly fewer than the normal data. The situations lead to some challenges for fault diagnosis in CPSs: the first one is the complexity of modern industrial systems sets’ obstacles for devising a practical model, and the features extracted from multi-dimensional sensor data depend too much on the experts’ knowledge; thus, handcrafted features influence the performance of fault diagnosis; the second one is the imbalance between faulty and normal samples, which has a serious impact on the performance of classifiers, for the reason that learning algorithms without consideration of imbalance tend to be overwhelmed by the majority class and ignore the minority class [2]. Some notable work [3,4,5,6,7] has been studied for industrial fault diagnosis based on machine learning algorithms; however, they have not taken the restrictions of these challenges into consideration. Consequently, a new model with automatic feature extraction and class-imbalance learning mechanism is necessary for the fault diagnosis.

Deep learning models have proven to be good methods for automatic feature extraction, so several deep learning methods to deal with sequential data are surveyed. Some papers have tried to use convolutional neural networks (CNN) [8] to recognize high-level patterns from multi-dimensional time series. CNNs have two main features: weights sharing and spatial pooling, which makes it very suitable for computer vision applications [9] whose inputs are usually two-dimensional data, and it has been also used to address natural language processing and speech recognition tasks whose inputs are 1-dimensional data [10,11]. However, to learn temporal and sequential features from time series better, the Long-Short Term Memory (LSTM) recurrent neural network has been utilized for high-level pattern recognition [12], as well as value prediction [13]. Furthermore, some hybrid models [14] combining CNN with LSTM have also been proposed to attain classification of time series by encoding both spatial and temporal features. Though many of the deep learning models mentioned above have achieved pretty good results in pattern recognition tasks, there is still plenty of room for improvement by considering temporal features among high-level patterns in a sequential style.

Considering the imbalance is another challenge in industrial fault diagnostic applications, several approaches for classification using imbalanced data have been surveyed. Under-sampling and over-sampling [15] can help with balancing data with sampling policies [16,17], which improve the distributions of features among majority and minority classes. SMOTE [18] is a synthetic technique that can enhance minority classes’ distributions among all samples. Chan et al. [19] introduce an approach to explore majority class examples, they split the majority class into several non-overlapping subsets, and finally ensemble classifiers using stacking. Xu et al. [20] propose EasyEnsemble and BalanceCascade algorithms to overcome the the difficulties in the class-imbalance learning problem. Cost-sensitive methods [21], which use cost functions to bias and optimize the misclassification loss, are also studied. Accordingly, how to use these class-imbalance learning strategies to optimize the deep learning models tends to be studied for industrial fault diagnosis.

Consequently, this paper proposes a weighted Long-term Recurrent Convolutional LSTM Network (wLRCL-D) for time series classification and imbalanced fault prediction. wLRCL is a hybrid deep representation learning model that composes with under-sampling policy to balance original imbalanced samples, a stacked Convolutional LSTM component to learn features inside subsequences, which represent high-level patterns, and another LSTM component to learn external temporal features among high-level patterns. Moreover, the loss function is optimized with weights on misclassification of imbalanced faulty classes. The contributions of this paper are summarized below:

1. A novel deep learning framework is proposed to learn both internal and external features of high-level patterns in an end-to-end way.
2. Data-level sampling policies and weighted loss function are integrated to the deep learning model to optimize the imbalanced fault classification.
3. The model is evaluated on a real-life datasets and proves its feasibility and effectiveness.

The remainder of the paper is organized as follows: in Section 2, several state-of-the-art deep learning models used for time series classification and prediction, and class-imbalance learning methods are surveyed at first. Then, the problem formulation is put forward to model the imbalanced fault diagnosis problem. Section 3 introduces the overview system and wLRCL-D model in detail. In Section 4, we present the comparable results between our model and other deep learning models in imbalanced fault prediction tasks based on PHM 2015 Challenge datasets. Finally, conclusions are drawn and future works are presented in Section 5.

## 2. Problem Statement

### 2.1. Deep Learning and Class-Imbalance Learning

#### 2.1.1. CNN, LSTM and DeepConvLSTM

**CNNs** can extract features from data automatically and a CNN with a single layer extracts features from the input signal through a convolution operation of the signal with a filter (or kernel). In a CNN, the activation of a unit represents the result of the convolution of the kernel with the input signal. These activated units filtered by the same kernel share the same parameterization (weight vector and bias) and form a feature map. By computing the activation of a unit on different regions of the same input (using a convolutional operation), it is possible to detect patterns captured by the kernels, regardless of where the pattern occurs. In CNNs, the kernels are optimized as part of the supervised training process, in an attempt to maximize the activation level of kernels for subsets of classes. The application of the convolution operator depends on the input dimensionality. With a temporal sequence of 2D images (e.g., a video), often 2D kernels are used in a 2D spatial convolution [22]. With a one-dimensional temporal sequence (e.g., a sensor signal), often a 1D kernel is used in a temporal convolution [23]. In the 1D domain, a kernel can be viewed as a filter, capable of removing outliers, filtering the data or acting as a feature detector, defined to respond maximally to specific temporal sequences within the timespan of the kernel. Formally, extracting a feature map using a one-dimensional convolution operation is given by:

(1) $$a_{j}^{l+1}\left(\tau \right)=\sigma \left(b_{j}^{l}+\sum_{f=1}^{F^{l}}K_{jf}^{l}\left(\tau \right)*a_{f}^{l}\left(\tau \right)\right)=\sigma \left(b_{j}^{l}+\sum_{f=1}^{F^{l}}\left[\sum_{p=1}^{P^{l}}K_{jf}^{l}\left(\tau \right)*a_{f}^{l}\left(\tau -p\right)\right]\right),$$

where $a_{j}^{j}\left(\tau \right)$ denotes the feature map *j* in layer *l*, σ is a nonlinear function, $F_{l}$ is the number of feature maps in layer *l*, $K_{jf}^{l}$ is the kernel convolved over feature map *f* in layer l to create the feature map *j* in layer (l+1), $P_{l}$ is the length of kernels in layer *l* and $b_{l}$ is a bias vector.

**LSTMs** extend RNN with memory cells, instead of recurrent units, to store and output information, easing the learning of temporal relationships on long time scales. LSTMs make use of the concept of gating: a mechanism based on component-wise multiplication of the input, which defines the behavior of each individual memory cell. The LSTM updates its cell state, according to the activation of the gates. The input provided to an LSTM is fed into different gates that control which operation is performed on the cell memory: write (input gate), read (output gate) or reset (forget gate). The activation of the LSTM units is calculated as in the RNNs. The computation of the hidden value $h_{t}$ of an LSTM cell is updated at every time step *t*. The vectorial representation (vectors denoting all units in a layer) of the update of an LSTM layer is as follows:

(2) $$i_{t}=\sigma_{i}\left(W_{ai}a_{t}+W_{hi}h_{t-1}+W_{ci}c_{t-1}+b_{i}\right),$$

(3) $$f_{t}=\sigma_{f}\left(W_{af}a_{t}+W_{hf}h_{t-1}+W_{cf}c_{t-1}+b_{f}\right),$$

(4) $$c_{t}=f_{t}c_{t-1}+i_{t}\sigma_{c}\left(W_{ac}a_{t}+W_{hc}h_{t-1}+b_{c}\right),$$

(5) $$o_{t}=\sigma_{o}\left(W_{ao}a_{t}+W_{ho}h_{t-1}+W_{co}c_{t}+b_{f}o\right),$$

where *i*, *f*, *o* and *c* are, respectively, the input gate, forget gate, output gate and cell activation vectors, all of which are the same size as vector *h* defining the hidden value. Terms σ represent nonlinear functions. The term $a_{t}$ is the input to the memory cell layer at time *t*. $W_{ai}$, $W_{hi}$, $W_{ci}$, $W_{af}$, $W_{hf}$, $W_{cf}$, $W_{ac}$, $W_{hc}$, $W_{ao}$, $W_{ho}$ and $W_{co}$ are weight matrices, with subscripts representing from-to relationships ($W_{ai}$ being the input-input gate matrix, $W_{hi}$ the hidden-input gate matrix, and so on). $b_{i}$, $b_{f}$, $b_{c}$ and $b_{o}$ are bias vectors.

**DeepConvLSTM** [24] is a kind of DNN, which comprises convolutional, recurrent and softmax layers. The convolutional layers act as feature extractors and provide abstract representations of the input sensor data in feature maps. The recurrent layers model the temporal dynamics of the activation of the feature maps. In this framework, convolutional layers do not include a pooling operation. The shorthand description of this model is: C(64)−C(64)−C(64)−C(64)−L(128)−L(128)−Sm, where $C(F^{l})$ denotes a convolutional layer *l* with $F^{l}$ feature maps, $R(n^{l})$ a recurrent LSTM layer with $n^{l}$ cells and Sm a softmax classifier. The input is processed in a layer-wise format, where each layer provides the representation of the input that will be used as data for the next layer. The number of kernels in the convolutional layers and the processing units in the dense layers is the same for both cases. The input to the network consists of a data sequence. The sequence is a short time series extracted from the sensor data using a sliding window approach composed of several sensor channels. The number of sensor channels is denoted as *D*. Within that sequence, all channels have the same number of samples $S^{1}$. The length of feature maps $S^{l}$ varies in different convolutional layers. The convolution is only computed where the input and the kernel fully overlap. Thus, the length of a feature map is defined by:

(6) $$S^{l+1}=S^{l}-P^{l}+1,$$

where $P^{l}$ is the length of kernels in layer *l*. The length of the kernels is the same for every convolutional layer, being defined as $P^{l}=5,\forall l=2,\ldots ,5$.

#### 2.1.2. Class-Imbalance Learning

An imbalanced dataset can be described as a set of samples, in which the proportion of the representative samples of one class is significantly larger than other classes. The amount of this proportion brings up the definition of the “imbalance ratio”, which is an important factor in selecting a proper classification technique. The imbalance ratio indicates that the collected data are highly imbalanced, moderate or low. The major class in an imbalance dataset referred to a class with more numbers of samples, while the minor class is often the class of interest and should be detected with high accuracy. In the industrial process, the faulty samples are usually minor classes compared with normal samples with different imbalance ratios.

To deal with the class-imbalance problem, two main strategies are usually used: data-level and algorithm-level methods. **Data-level** method [25,26] is to change the class distribution of imbalanced data by sampling policies. Under-sampling and over-sampling [27] are two common methods. Ref. [27] indicates that the random under-sampling RUS strategy usually outperformed some other complicated under-sampling strategies. In addition, SMOTE [26] is a synthetic over-sampling technique, which can added new minority class examples. Han et al. [28] proposed the borderline-SMOTE to over-sample the minority class near the borderline. Xie at al. [29] showed that over-sampling methods usually perform better than under-sampling methods. Estabrooks et al. [30] and Barandela et al. [31] both suggested that a combination of over-sampling and under-sampling might be more effective to solve the class imbalance problem. However, it is argued that the sampling method leads to overfitting or dropping some useful features of majority classes. The **algorithm-level** method [25] is to adjust the classifier to imbalance data. The bagging and boosting ensemble-based method have been widely used. Seiffert et al. [32] conducted a comprehensive study comparing sampling methods with boosting for improving the performance of a decision trees model built for identifying the software defective modules. Their results showed that sampling methods were effective in improving the performance of such models while boosting outperformed even the best data sampling methods. Chawla et al. [33] proposed a novel approach SMOTEBoost for learning from imbalanced datasets on the basis of the SMOTE algorithm and the boosting procedure. Seiffert et al. [34] presented a different hybrid ensemble methods named RUSBoost, which combined the random under-sampling strategy with the boosting procedure. Ref. [35] proposed the EasyEnsemble method, which changes the imbalance learning problem into several balance classification tasks with ensemble strategies. The idea is based on a twofold number of ensembles that under-samples the majority class without information loss and adaboost [36] is used to train the weak classifiers. Cost-sensitive methods either use different misclassification costs associated with each class sample (i.e., data-level approach) or alter the training procedures to take costs (i.e., algorithm-level approach), in order to bias the classifier toward the rare class [21]. Ref. [37] showed that the incorporation of costs in the error function improves performance. Refs. [38,39,40] incorporate class-specific costs in the deep networks. Ref. [38] proposed a new CoSen loss function, which replaces traditional softmax with a regression loss.

### 2.2. Problem Formulation

**Figure 1:**
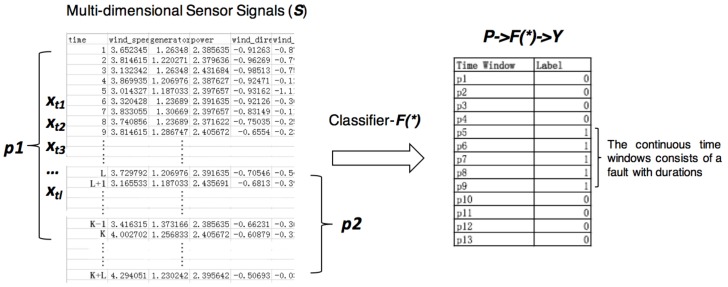
Formulate machine fault diagnostic problems into classification problems: segment the multi-dimensional sensor signals into time windows with fault type labels.

A diagnostic signals collected from machines are multi-dimensional time series, which is a sequence of real-valued data points with timestamps generated by *D* different sensor channels. Thus, the raw data $x_{ti}$ at any timestamp *i* is a multidimensional vector that can be described as a tuple $\left(t_{i},\vec{V_{i}}\right)$, where $t_{i}$ is a timestamp and $\vec{V_{i}}\in R^{D}$ is a D-dimensional vector of measurements. Given a original signals from a machine $S=\{x_{0},x_{1},\ldots ,x_{ti}\}$, the targets of diagnosing the machine are to recognize whether it is at failure state and determine the fault type at given time $t_{i}$, as well as its durations. In this paper, we focus on how to recognize fault types and the problem can be formulated into a classification problem based on machine learning models. As Figure 1 shows, the original signals *S* are segmented into subsequences *P* by sliding windows, and each subsequence belongs to a normal or faulty event with corresponding labels *Y*. To diagnose the whole sequence is based on recognizing each one of its subsequences with a classifier F(∗). We denote the input of the classifier as $P=\{p_{1},p_{2},\ldots ,p_{n}\}$, where $p_{i}=\left\{x_{t1},x_{t2},\ldots ,x_{tl}\right\}$ represents a subsequence. The $x_{ti}$ is the value at time stamp *i* in the current time window, and there are *l* time stamps in each $p_{i}$. The corresponding output is $Y_{i}=\left\{y_{1},y_{2},\ldots ,y_{n}\right\}$. For ease of representation, in this paper, we consider classification problems, where $y_{i}$ is a categorical value in C={1,…,C} and where $C\in Z^{+}$ is the number of classes including different types of faults and normal event. Consequently, fault diagnostic tasks are formulated as training a classifier F(∗) that could predict the right outputs as many as possible.

In this paper, it is aimed to design a method that is able to automatically extract and learn features of imbalanced faulty and normal samples with F(∗). Three strategies are considered to be used:

1. Deep learning model is suitable to automatically extract features from raw multi-channel sensor data, with both spatial and temporal features. Subsequences of original time series signals represent high-level patterns of the observed object, while the whole time series represent its temporal evolutions, so new deep learning models proposed in this paper should focus on capturing the spatial and temporal features inside a subsequence, as well as temporal features between subsequences. In our model, the sliding window of fixed length *l* with a step of l/2 is proposed to segment the original signal into subsequences;
2. The data-level method is necessary to balance the faulty subsequences and normal subsequences with under-sampling and over-sampling policies. Sampling is the most straightforward method, which makes the imbalanced samples relatively balanced before training by a classifier. Thus, considered faulty samples are at a high imbalance ratio, and an under-sampling preprocess can be used to decrease the imbalance ratio before training the classifier in this paper;
3. The algorithm-level method is necessary to optimize the baseline classifier to better adjust distributions of imbalanced faulty classes. Sampling methods sometimes lead to a distortion of feature distribution for both majority and minority classes. Thus, a weighted cost-sensitive methods can be used in this paper. Let W(i|j) be used to denote the weights on misclassification cost h(j|x) of classifying an instance belonging to a class *i* into a different class *j*. Given an input instance *x* and the weight matrix W(i|j), the classifier seeks to minimize the expected loss function H(i|x) as Equation (7) shows, where *i* is the class prediction made by the classifier:
  (7) $$\begin{array}{cc} p^{*} & =\underset{i}{\arg\min}H\left(i|x\right)=\underset{i}{\arg\min}\left(\sum_{j\in (-,+)}W\left(i|j\right)h\left(j|x\right)\right)\\ & =\underset{i}{\arg\min}\left(-\frac{1}{N}W\left(i|j\right)\sum_{i}^{N}\sum_{j}^{M}y_{j}\cdot \ln\left(P(j|x)\right)\right)=\underset{i}{\arg\min}\left(\sum_{j}^{M}\left(-\frac{1}{N}W\left(i|j\right)\sum_{i}^{N}y_{j}\cdot \ln\left(P(j|x)\right)\right)\right), \end{array}$$
  where h(j|x) is the cross-entropy loss of classifying *x* into class *j*, *N* is the total number of training samples, *M* is the total number of classes, $y_{j}$ is the predicted class and P(j|x) is the posterior probability over class *j* given an instance x.

## 3. System Model

### 3.1. Pipeline Overview

**Figure 2:**
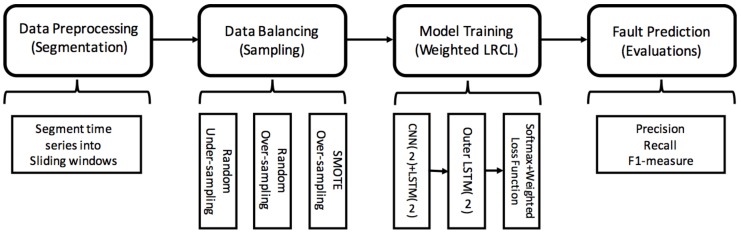
System model: pipeline overview of processing imbalanced fault diagnosis.

The pipeline of processing the imbalanced fault diagnosis is composed of four main steps as Figure 2 shows: (1) **Data preprocessing:** segment the multi-dimensional sensor data into time windows and each segment is labeled with fault types or normal event; (2) **Data balancing:** balance the faulty segments and normal segments with under-sampling and over-sampling policies. Under-sampling is used to sample normal samples, such as random under-sampling, while over-sampling is to enhance faulty samples, such as random under-sampling and SMOTE; (3) **Model training:** train a deep-learning-based imbalance-class classifier. A weighted Long-term Recurrent Convolutional LSTM network is proposed, which consists of a 2-layer CNN stacked 2-layer inner LSTM to learn the internal features of a time window segment, a 2-layer outer LSTM to learn the temporal features among segments, and a softmax layer for multi-class classification. The loss function is a class-imbalance weighted loss function that takes the weight of minority and majority classes into consideration; and (4) **Fault prediction:** predict faulty segments with the trained model and evaluate the performance with precision, recall and F1-measure.

### 3.2. Weighted Long-Term Recurrent Convolutional LSTM Network

**Figure 3:**
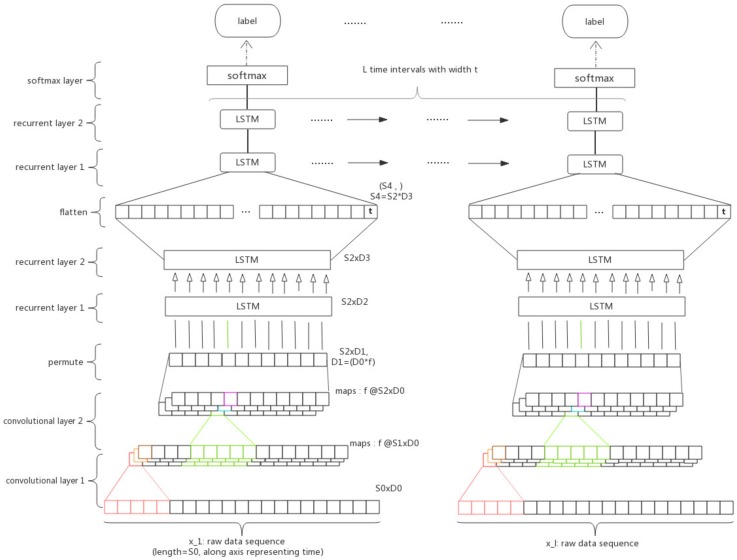
Main architecture of the LR-ConvLSTM model.

The weighted Long-term Recurrent Convolutional LSTM(wLRCL) has three major components as Figure 3 shows: the convolutional layers, the inner LSTM layers and the outer LSTM layers, stacked from bottom to top, and the statement of the inner and outer recurrent layers individually refers to capturing features from the sensor sequence in sliding windows and from the sequence of states.

**Convolutional layers:** each of the subsequence $p_{i}$ is fed into the convolutional layers and the output are the filtered features of the subsequence via 2-layered CNNs. The extracted features represent the spatial and short-term temporal features of the multi-dimensional signals inside a segmented time window. Since the structures of individual convolutional subnets for different subsequences are the same, one individual convolutional subnet with the input $p_{i}$ is primarily introduced. According to the definition in Section 2.2, $p_{i}=\left\{x_{t1},x_{t2},\ldots ,x_{tl}\right\}$, and the input $p_{i}$ formulates a $S_{0}\times D_{0}$ tensor, where $S_{0}$ is equal to the sequence length of sliding window *l*, and $D_{0}$ is equal to the number of sensor channels *D*. For each time interval *l*, the matrix $p_{i}$ will be fed into a CNN architecture. 1D filters with shape (k,1) are used to learn the spatial and short-term temporal dynamics from the input $p_{i}$. In this paper, the size of the filters in every convolutional layer is the same, and the convolution is only computed where the input and the filter fully overlap. For each convolutional layer, the model learns *f* filters, through which the model obtained more nonlinear functions and learned more global information of the current sequence, and use ReLU as the activity function. The convolutional layer is not followed by a pooling operation, as the next recurrent layers require a data sequence to process. The shape of feature maps output by the *m* convolutional layers is $S_{m}\times D_{0}$, where $S_{m}=S_{0}-m\left(k-1\right)$ is the new sequence length of the time window.

**Inner LSTM layers:** a 2-layered inner LSTMs are stacked on the CNNs to learn the internal long-term temporal features of each subsequence. In order to make the feature maps processed by the former convolutional layers conform to the input format of the LSTM, we need to permute the data here. Assuming that the shape of the output of CNNs is $\left(f,S_{m},D_{0}\right)$, we need to change it to $\left(S_{m},f\times D_{0}\right)$ as the input of the first LSTM layer. Thus, the length of input of the LSTM is $S_{m}$, and the dimension of input is $D_{1}$, where $D_{1}=f\times D_{0}$. After the operation of 2-layered LSTMs, our model has captured the long-term dependencies from the sliding window and output a matrix of shape of $\left(S_{m},D_{2}\right)$. Then, we flatten the output matrix into vector $V_{i}$, which can be seen as the feature representation of a high level pattern or state.

**Outer LSTM layers:** an outer LSTM is stacked on the CNNs and inner LSTMs to learn the long-term temporal features among each subsequence. As a result of the operation of convolutional and inner recurrent layers, the sensor data $x_{i}=\left\{t_{1},t_{2},\ldots ,t_{w}\right\}$ in a sliding window has been processed to a feature vector $V_{i}$. Concatenating all *l* vectors $\left\{V_{1},V_{2},\ldots ,V_{l}\right\}$ into a *l*-row matrix V, which is the input of the recurrent layers. Then, the output of LSTMs at every time step is fed into a softmax logistic regression output layer, which yields the classification outcome $y_{i}$. Thus, the input of this part of LSTM is V, and the output is Y.

**Class-balanced weighted loss function:** the output layer is a softmax layer, which is usually used for multi-class classification. According to Equation (7), the loss function of the classifier could be weighted by the weight matrix W(i|j), which represents the weights on misclassification cost h(j|x) of classifying an instance belonging to a class *i* into a different class *j*. To optimize the loss function that is able to adjust the imbalance between faulty classes and normal class, the weight W(i|j) is redesigned according to the imbalance ratio. It is assumed that the majority class with more features learned has less cost of misclassifications and minority classes with fewer features learned have more cost of misclassifications. Thus, we define the weight W(i|j) of each cross-entropy loss as the inverse ratio of imbalance ratio of misclassified class *j*, which is denoted as $W\left(i|j\right)=\frac{N}{N_{j}},$ where *N* is the total number of training samples and $N_{j}$ is the total number of samples belonging to class *j* in training data. The class-imbalanced weighted loss function is defined as Equation (8) shown:

(8) $$H\left(i|x\right)=\sum_{j}^{M}\left(-\frac{1}{N}\cdot \frac{N}{N_{j}}\sum_{i}^{N}y_{j}\cdot \ln\left(P(j|x)\right)\right)=\sum_{j}^{M}\left(-\frac{1}{N_{j}}\sum_{i}^{N}y_{j}\cdot \ln\left(P(j|x)\right)\right).$$

## 4. Experiments and Results

### 4.1. Data Preparation and Experiment Settings

**Figure 4:**
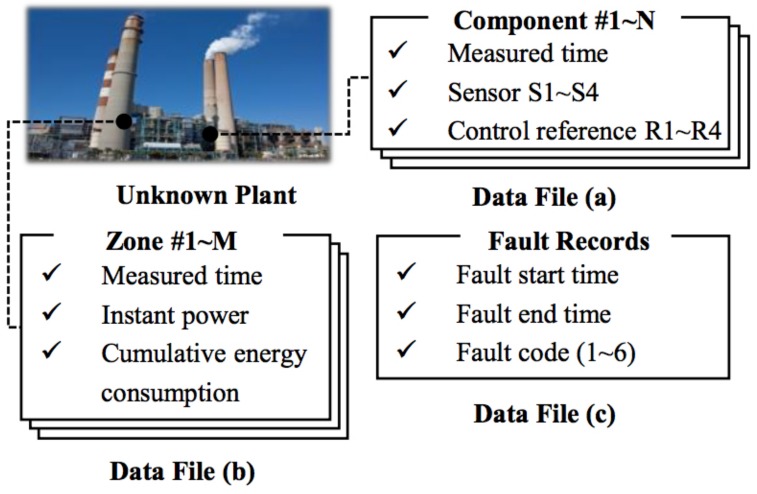
Descriptions of the datasets.

The dataset is provided by PHM 2015 Challenge (PHM 2015 Challenge: https://www.phmsociety.org/events/conference/phm/15/datachallenge), which records the actual working conditions of several industrial plants, including six kinds of faults as well as normal events. The datasets consist of the following three parts (shown in Figure 4):

1. time series of sensor measurements and control reference signals for each of a number of control components of the plant (e.g., six components);
2. time series data representing additional measurements of a fixed number of plant zones over the same period of time (e.g., three zones), where a zone may cover one or more plant components;
3. plant fault events, each characterized by a start time, an end time, and a failure code.

Only faults of types 1–5 are of interest, while code 6 represents all other faults not in focus. The frequency of measurements is approximately one sample every 15 minutes, and the time series data spans a period of approximately three to four years. The goal of the challenge is to predict the beginning time and end time of failure events of types 1–5. In our case, we formulate it as a imbalanced fault classification problem and only focus on the prediction of the failure mode without the consideration of the failure start and end time. The dataset can be downloaded from NASAAmes Prognostics Data Repository [41].

To better evaluate the results, plant #1’s training datasets with labels are used to validate the performance of our model on imbalanced fault classification. Firstly, the original data are segmented into time windows with step lengths of 12, each of which is with 48 dimensions (six machines with eight dimensions for each), and each time window is labeled with fault and normal types. Then, the original labeled data are divided into training dataset and test dataset with 9:1 proportion. Initial statistics of the imbalance ratio are given in Table 1, and the results show that each type of fault is at a different imbalance ratio.

In our case, we choose 24, 48 and 100 as the time window length. Then, we compare several baseline methods as follows. In Table 2, the suffix of each methods is listed, and the sampling rates for RUS and SMOTE are illustrated. The source code of wLRCL-D has been open-sourced on github (wRCL-D source code: https://github.com/minelabwot/DeepLearning_WoT).

1. **XGBoost** (abbreviated as XGB): It uses the entire dataset (P and N) to train an ensemble classifier. The number of iterations is 5000.
2. **EasyEnsemble+SMOTE+XGBoost** (abbreviated as Easy-SMT): Number of subsets $T_{1}=4$, for each subsets $N_{i}$, we generate $P^{\prime }$ using SMOTE, a set of synthetic minority class examples with $\left|P^{\prime }\right|$ = $\left|N_{i}\right|$ − $\left|P\right|$. Then, XGBoost is used to train a classifier using $P+P^{\prime }$ and $N_{i}$. The number of iteration is 5000.
3. **CNN-D**: four CNN layers with random under-sampling.
4. **DeepConvLSTM-D**: four CNN layers stacked with two LSTM layers, as well as random under-sampling policy.
5. **LRCL-O**: two CNN layers and two inner LSTM layers stacked with two outer LSTM layers, as well as SMOTE-based over-sampling policy.
6. **LRCL-W** (abbreviated as wLRCL): two CNN layers and two inner LSTM layers stacked with two outer LSTM layers, as well as weight-based cost-sensitive policy.
7. **LRCL-D**: two CNN layers and two inner LSTM layers stacked with two outer LSTM layers, as well as random under-sampling policy.
8. **LRCL-D-W** (abbreviated as wLRCL-D): two CNN layers and two inner LSTM layers stacked with two outer LSTM layers, as well as random under-sampling and weight-based cost-sensitive policies.

### 4.2. Results and Evaluations

**Figure 5:**
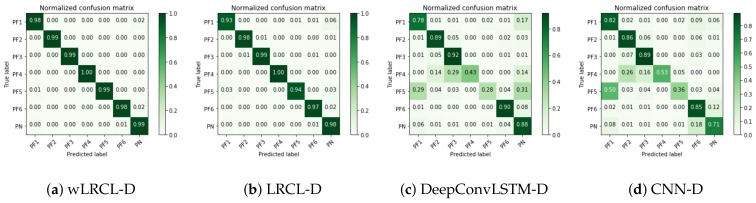
Confusion matrix of wLRCL-D (**a**); LRCL-D (**b**); DeepConvLSTM-D (**c**) and CNN-D (**d**).

As Table 3 shows, the wLRC-D outperforms other baseline methods in average precision, recall and F1 evaluation metrics based on window lengths of 24, 48 and 100. In detailed discussions, firstly, compared with LRCL, wLRCL and XGBoost, it can be found that LRCL and XGBoost achieve similar performance as baseline classifiers, which illustrates that the deep learning method does not perform much better than the ensemble method on imbalanced fault classification tasks, while performance has been improved a lot via wLRCL, which illustrates that weighted loss function optimizes the imbalanced fault classification compared to LRCL. Secondly, comparing CNN-D, Easy-SMT, LRCL-O, DeepConvLSTM-D, LRCL-D, wLRCL-D with XGBoost, LRCL and wLRCL, it can be found that sampling methods (whether under-sampling or over-sampling) increase the classification performance compared to the baseline classifiers without sampling policies. Thirdly, comparing wLRCL-D, LRCL-D, DeepConvLSTM-D, LRCL-O with EasySMT, it can be found that deep learning methods achieve better classification performance than the non-deep learning method based on sampling policies. Finally, compared with wLRCL-D, LRCL-D, DeepConvLSTM-D, LRCL-O and CNN-D, it can be found that LRCL is better than DeepConvLSTM and CNN on feature representation learning of multi-channel sensor data, since the temporal features among time window segments have been learned by LRCL. Then, random under-sampling policy has more positive effects than SMOTE-based over-sampling policy on LRCL classifiers by comparing it with LRCL-D and LRCL-O. In addition, weighted loss function achieves further improvements on imbalanced fault classification task of LRCL with under-sampling policies. To better understand how these models classify each fault type, Figure 5 presents the confusion matrix of wLRCL-D, LRCL-D, DeepConvLSTM-D and CNN-D. The results show that wLRCL-D performs best in recognizing each of the fault types, and LRCL-D without weighted adaptation is worse than wLRCL-D but is quite close to it, while DeepConvLSTM-D and CNN-D perform worse than wLRCL-D and LRCL-D on all classes, especially on recognizing fault types 4 and 5, which have the two most imbalanced ratios. In general, our proposed method wLRCL-D has achieved significant performance improvement compared to other baseline methods on imbalanced fault diagnostic tasks of the PHM 2015 Challenge.

**Figure 6:**
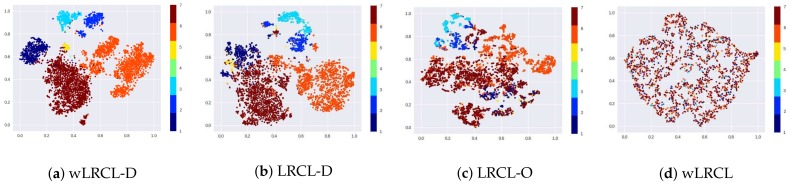
t-SNE cluster map of wLRCL-D (**a**); LRCL-D (**b**); LRCL-O (**c**) and wLRCL (**d**).

To better understand the feature representation learning of different LRCL-based deep learning models, the t-SNE cluster maps are presented in Figure 6. The results illustrate that wLRCL-D outperforms most other LRCL-based methods on learning feature representations of different types of faults, since weighted cost-sensitive and under-sampling methods optimize the distributions of imbalanced faulty samples jointly. Comparing LRCL-O with LRCL-D, it is obvious from the results that under-sampling of normal samples achieves more optimizations than SMOTE-based over-sampling of faulty samples. In addition, the results also show that sampling-based methods perform better than weighted cost-sensitive methods by comparing LRCL-O and LRCL-D with wLRCL.

**Figure 7:**
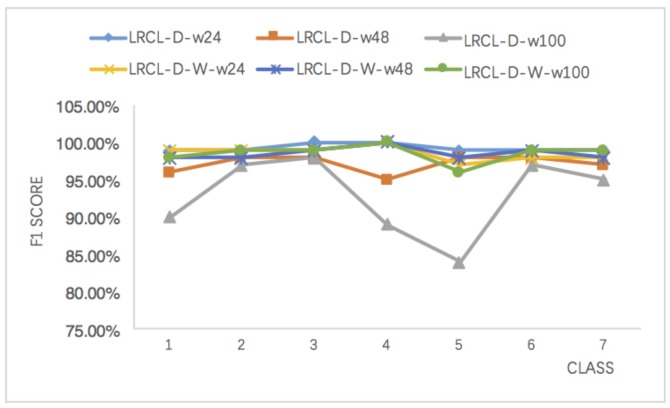
The influence of window length on LRCL-D and wLRCL-D.

Moreover, the effects of the window length on wLRCL-D’s performance have been evaluated as Figure 7 and Table 4 show. In Table 4, the F1 of LRCL-D and wLRCL-D with window lengths 24, 48 and 100 have been shown respectively. Firstly, the results illustrate that F1 score fluctuates a lot for LRCL-D with different window lengths, and the longer the window length, the worse the fault classification performance, while the variation of window length on wLRCL-D has little influence on the classification performance, which means that wLRCL-D is more robust and stable to learn the segments’ features with different lengths than LRCL-D.

In Table 4, the F1 and Recall of wLRCL and wLRCL-D with window lengths 24, 48, and 100 have been shown respectively. As an overview, it is obvious that the performance is more robust for wLRCL-D than wLRCL with different window lengths. The result illustrates that window length is an important hyper-parameter, which influences the performance of wLRCL, and random under-sampling policy is able to alleviate the dependency on the length of time windows for wLRCL’s performance. In detailed discussions, the sliding window represents a sampling process of segmented features from total sequences, and different window lengths cover different feature spaces of faulty samples. Thus, shorter window length may not cover sufficient features of majority samples, while longer window length perhaps ignores some useful features of minority samples. All of these situations will make the classifier not able to learn the features well among imbalanced samples, and it requires a suitable window length choice before training the model without any sampling policies. On the other hand, the under-sampling of normal samples makes the data relatively balanced, and it makes the features not sensitive to the variations of window lengths by classifiers.

**Figure 8:**
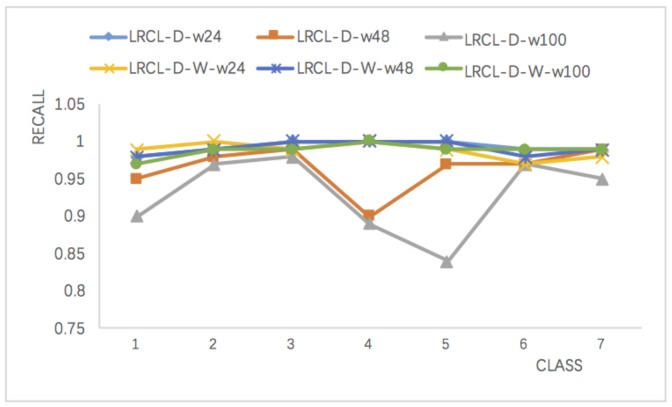
Comparisons of recall values on LRCL-D and and wLRCL-D with different window lengths.

Recall is suitable for measuring minority class recognition performance and Figure 8 compares LRCL-D and wLRCL-D with different window lengths. The results show that wLRCL-D performs better than LRCL-D on faults (class type 1–5) classifications, especially on fault types 4 and 5. Fault types 4 and 5 are the two most imbalanced faults, and the results illustrate that wLRCL-D is more robust on extremely imbalanced datasets. The reason can be deduced that the weighted loss function rewards the cost of classifying minority class and punishing the cost of classifying majority class.

**Figure 9:**
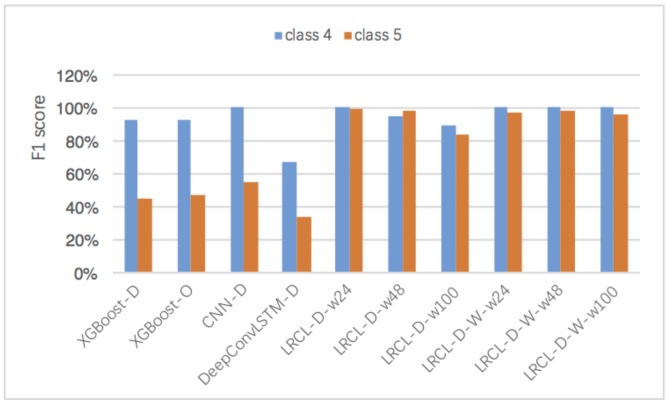
Comparisons of F1 score on classifications of faults 4 and 5 based on different methods.

To further evaluate the performance of recognition on minority classes, we compare the F1 scores on the classification of faults 4 and 5 in Figure 9, which takes up 0.06% and 0.65%, respectively. The results show that LRCL methods with sampling or weighted policies perform better than XGBoost and CNN on both faults 4 and 5. In addition, wLRCL-D with different window lengths performs better than wLRCL, which illustrates that wLRCL is more robust for different window lengths with under-sampling policy.

## 5. Conclusions

This paper proposes an efficient weighted deep representation learning model (wLRCL-D) for imbalanced fault diagnosis in CPS. The deep learning model contains 2-layer CNN and 2-layer inner LSTM to learn the internal spatial and temporal features inside a time window segment, and 2-layer outer LSTM to learn the external temporal features among time window segments. In addition, random-under sampling is used to balance the training samples, and a weighted loss function is designed to optimize the imbalance data distributions. This model has been evaluated on PHM 2015 challenge datasets. The achieved results show that wLCRL-D outperforms other baseline classifiers in precision, recall and F1 scores. The experiments suggest that wLCRL-D is efficient and robust feature learning in addition to the imbalanced fault diagnostic approach based on the deep learning method. In future work, a more adaptive cost-sensitive weight mechanism can be studied based on the loss function of deep neural networks to adjust different imbalance ratios of data.

## Figures and Tables

**Table 1:** The ratio of faults and normal events in plant #1. PF means plant faulty samples and PN means plant normal samples.

Event Type	PF1	PF2	PF3	PF4	PF5	PF6	PN
Ratio	4.83%	3.75%	3.39%	0.06%	0.65%	19.26%	68.06%

**Table 2:** The suffix of methods used based on baseline classifiers.

Suffix	Description	Parameters
-W	Cost-sensitive Weight Method	—–
-O	SMOTE-based Method	All faulty classes are over-sampled to 5000
-D	Random Under-sampling Method	Normal class is under-sampled to 10,000

**Table 3:** Comparisons of average precision, recall and F1 among different methods based on 24, 48 and 100 time window lengths.

Method	Precision	Recall	F1
XGBoost	53.57%	59.81%	56.02%
LRCL	51.92%	64.31%	55.36%
wLRCL	66.80%	75.04%	69.87%
CNN-D	80.86%	80.88%	80.81%
Easy-SMT	84.19%	84.38%	84.0%
LRCL-O	84.94%	89.23%	86.95%
DeepConvLSTM-D	88.48%	88.37%	88.40%
LRCL-D	95.51%	97.30%	97.29%
wLRCL-D	98.42%	98.46%	98.46%

**Table 4:** The influence of window length on wLRCL and wLRCL-D.

Window_length	24	48	100
F1(wLRCL)	56.66%	99.51%	54.42%
Recall(wLRCL)	64.23%	99.51%	61.38%
F1(wLRCL-D)	98.24%	98.40%	98.75%
Recall(wLRCL-D)	98.24%	98.40%	98.75%
